# Parental Burnout and Child Behavior: A Preliminary Analysis of Mediating and Moderating Effects of Positive Parenting

**DOI:** 10.3390/children11030353

**Published:** 2024-03-16

**Authors:** Aline Woine, María Josefina Escobar, Carolina Panesso, Dorota Szczygieł, Moïra Mikolajczak, Isabelle Roskam

**Affiliations:** 1 Psychological Sciences Research Institute, Department of Psychology, Catholic University of Louvain, Place Cardinal Mercier, 10, 1348 Louvain-la-Neuve, Belgium; aline.woine@uclouvain.be (A.W.); moira.mikolajczak@uclouvain.be (M.M.); 2 Center for Social and Cognitive Neuroscience, School of Psychology, Universidad Adolfo Ibañez, Santiago 7941169, Chile; mjosefina.escobar@uai.cl (M.J.E.); carolina.panesso@edu.uai.cl (C.P.); 3 Department of Psychology, Faculty in Sopot, SWPS University, 81-745 Sopot, Poland; dszczygiel@swps.edu.pl

**Keywords:** parental exhaustion, positive parenting, replicability, internalizing/externalizing behaviors

## Abstract

Despite its significant growth over the past fifteen years, research on parental burnout is just beginning to explore the relationships of the syndrome with child behavior. Previous research with adolescents has shown the existence of associations between parental burnout and internalizing and externalizing behaviors in the offspring. The current study is an attempt to (i) replicate this preliminary evidence specifically among Chilean preschool children and (ii) explore the mediating/moderating effects of positive parenting that may be involved in these putative associations. A sample of 383 Chilean mothers participated in this cross-sectional online study. The results confirmed the associations between parental burnout and child internalizing and externalizing behaviors. We also observed that positive parenting was a mediator in the relationship linking parental burnout and the child’s internalizing (full mediation) and externalizing (partial mediation) behaviors. Positive parenting also partially mediated the association between the child’s externalizing behavior and parental burnout. Our results further suggested that the child’s externalizing behavior was possibly a more substantial contributing factor to parental burnout than the child’s internalizing behavior.

## 1. Introduction

If the vast majority of today’s parents feel a sense of accomplishment in their role as parents, an important proportion of other parents nevertheless falter under the heavy burden of their parental role [1]. For no less than 5% of Western parents [2,3], their parental role has become so burdensome that they are exhausted, to the point of burning out.

Parental burnout, a syndrome characterized by intense exhaustion, results from a significant and prolonged stress associated with parenthood in the absence of sufficient resources for coping [4]. As suggested by the authors of the etiological model [4], none other than this long-lasting imbalance between parental resources and stressors is the mechanism responsible for parental burnout. The syndrome includes four core symptoms that occur in several phases, with the exhaustion phase appearing first [5] and subsequently giving way to the other symptoms, which reinforce each other to eventually form a dense network of interrelated symptoms [6]. First, the parent feels exhausted by parenting to the point of feeling unable to get out of bed to face another insurmountable day with the children. In a second instance, the parent experiences the irrepressible need to emotionally distance themselves from their children. Third, we observe that the parent has lost all pleasure in their parental role, which has become a real chore. Moreover, we can observe a sharp contrast between the now minimalist parent and the previously dedicated parent they once were [7].

As a recent meta-analysis on parental burnout shows [8], correlates associated with parental burnout have been extensively researched (albeit mainly cross-sectionally) over the past fifteen years. Studies have shown that the syndrome is associated with variables that fall primarily into three distinct categories. The first category relates to the personal traits/characteristics of the parents. Thus, the scientific literature has singled out factors such as—to name only the most prominent ones—low emotional competence [9], high perfectionist parenting concerns/aspirations [10,11] and parenting (see [12] for the association between parental burnout and parental hostility; see [9] for the association between parental burnout and parenting; see [13] for the association between parental burnout and the authoritarian parenting style).

The second category of correlates relates to family functioning characteristics, where an important degree of family disorganization (to a greater extent) [9,10] and co-parenting disagreement (to a lesser extent) [10] have been shown to be associated with parental burnout.

The third category of correlates relates to the child and specifically to either their characteristics or behavior. Previous studies have highlighted that having chronically ill children [14,15,16] or caring for children with special needs [17] is a correlate of parental burnout. Regarding child behavior, more recently, refs. [12,18] have shown an association between parental burnout and child externalizing (i.e., irritability, aggressiveness, oppositional defiant disorder [19]) and/or internalizing behaviors (i.e., somatic complaints, anxiety and depressive symptoms, suicidality [20]).

Thus, although the young field of parental burnout research has provided much insight into the correlates of the syndrome, we need more research focusing on how these correlates potentially interact with each other, particularly in terms of mediating and moderating processes (e.g., [12,21,22]. The present paper proposes to contribute to this effort by cross-sectionally examining the relationships between two specific correlates of parental burnout, namely child behavior (externalizing and internalizing) and positive parenting (i.e., “parental behavior based on the best interests of the child that is nurturing, empowering, non-violent and provides recognition and guidance which involves setting of boundaries to enable the full development of the child” (Council of Europe, 2006, p. 6)). Sanders [23,24] further identified five core principles of positive parenting that consider both protective and risk factors predicting positive developmental and mental health outcomes in children. They are providing the child with a safe and engaging environment, providing the child with a positive learning environment, applying assertive discipline, having realistic expectations and engaging in parental self-care. In terms of the implications of positive parenting on child behavior, past research showed that positive parenting reduces the impact on the child’s externalizing and internalizing behaviors [25,26].

Of the three categories of parental burnout correlates outlined above, the last one (that relates to the child’s characteristics or behavior) has received much less scholarly attention thus far. In the studies of the few authors who have addressed this issue, we observe associations between parental burnout and child externalizing and internalizing behaviors [12,18,27,28]. However, because these studies were conducted in specific contexts (i.e., in Chinese settings with adolescents for the first, third and fourth studies) and under specific conditions (i.e., in the COVID-19 pandemic for Kerr’s study with only five items from the Parental Burnout Assessment Scale (PBA, [29]) and using a subset of children’s health questions from the National Survey of Children’s Health (Child and Adolescent Health Measurement Initiative, 2018)), these associations need to be replicated in different age groups and in different cultural contexts both with the 23 items that comprise the Parental Burnout Assessment Scale (PBA, [29]) and with a measure specifically intended to assess the child’s internalizing and externalizing behaviors.

If the associations between parental burnout and child behavior were to be replicated, it is likely that they would not be direct. Rather, they may occur sequentially through mediating processes. The indirect effect assumption seems tenable since it is barely imaginable that a state of psychological distress on its own (such as parental burnout) can impact the child’s behavior, as such an abstract state of psychological distress is necessarily reflected in the subject’s concrete behaviors. It is precisely such concrete parental behaviors (represented in this study as poor positive parenting) that, in turn, could impact the child’s behavior. Likewise, the associations between parental burnout and child behavior may be moderated for the better (buffering effect)—or for the worse (exacerbating effect)—by moderating processes since, for example, not all burnt-out parents have a deleterious impact on their child’s behavior, or since, for example, not all unruly children lead their parent to burnout. We propose here to examine a potential mediator/moderator in this association for which the scientific literature has shown it to be importantly associated with parental burnout and/or child outcomes, namely positive parenting [25,26,30,31,32]. 

First, as regards the association between positive parenting and parental burnout, previous studies show that positive parenting is negatively correlated with parental burnout (*r* = −0.38 in [9]; see [13] for the association between authoritarian parenting practices and parental burnout). Second, as regards the association between positive parenting and child outcomes/behavior, a vast body of literature extensively documents that (poor) positive parenting has (detrimental) effects on child outcomes and behavior [33,34,35,36,37,38].

Since we are exploring the mediating/moderating effects of positive parenting in the bivariate association between parental burnout and child behavior, we feel it is crucial to conduct an exploratory test on the putative mediated/moderated effects of positive parenting in this association in both directions, that is, from parental burnout to child behavior and from child behavior to parental burnout.

Thus, shifting the focus specifically to the putative mediating effects, we propose to explore whether scoring low on positive parenting would constitute a potential mediator in the association between parental burnout and child behavior. Thus, for example, we propose that by being unruly or emotionally unstable, the child might create a stressful environment for the parent, which may lead the parent to engage in little positive parenting [39,40]. In turn, a parent who scores low on positive parenting might increase their parental burnout symptomatology. As for the reverse direction (since we are examining bivariate correlations that preclude any direction), we suggest that a burnt-out parent, due to their condition [6], may be less inclined to resort to positive parenting, which in turn might deleteriously affect their child’s behavior.

In addition to mediating processes, we propose to explore whether moderating processes would be at play in the association between parental burnout and child behavior. As previously stated, this assumed presence of moderators in this association seems obvious, since not all parents whose child displays externalizing and/or internalizing behaviors are necessarily burnt out, and similarly, not all children with a parent suffering from parental burnout necessarily develop externalizing and/or internalizing behaviors. Specifically, based on the above example, we assume that a parent who—regardless of the context or situation—would engage in (little) positive parenting would be less (more) prone to parental burnout if their child is unruly or emotionally unstable (buffering/aggravating effect of the parent’s positive parenting in the association between child behavior and parental burnout). Now considering the reverse direction—still independent of context but based on the specific individual characteristics of the burnt-out parent—the parent may/may not expose their child to the risk of externalizing and/or internalizing behaviors if they score low/high on positive parenting. This refers to the aggravating or buffering effect of the parent’s positive parenting in the association between parental burnout and child behavior.

The aim of the present cross-sectional study is twofold. First, it aims to examine whether the observed associations between parental burnout and the child’s externalizing/internalizing behaviors in Chinese settings with adolescents (correlations between parental burnout and child externalizing/internalizing behaviors range from 0.12 to 0.24 in [12,27,28]) can be replicated in our study conducted on Chilean mothers of preschool children. We will also examine the extent to which parental burnout and child behavior correlate in our sample (i.e., 0.10 = weak, 0.30 = moderate or 0.50 = strong [41]). These correlations will be compared with those obtained in [12,27,28], aiming to assess whether the association between parental burnout and child externalizing behavior is stronger than that between parental burnout and child internalizing behavior, as observed by [12]. Second, assuming that the hypothesized association between parental burnout and child behavior can be replicated, we will investigate the potential mediating/moderating effects of positive parenting on the bivariate associations. As in the case of the relationship between parental burnout and child behavior, very little literature sheds light on the mediating/moderating aspects in the association between parental burnout and child behavior (see [12,27,28] for the only attempts with mediation analysis among Chinese adolescents). Therefore, we cannot hypothesize whether positive parenting will mediate rather than moderate (or vice versa, or both) the association considered here.

**Figure 1:**
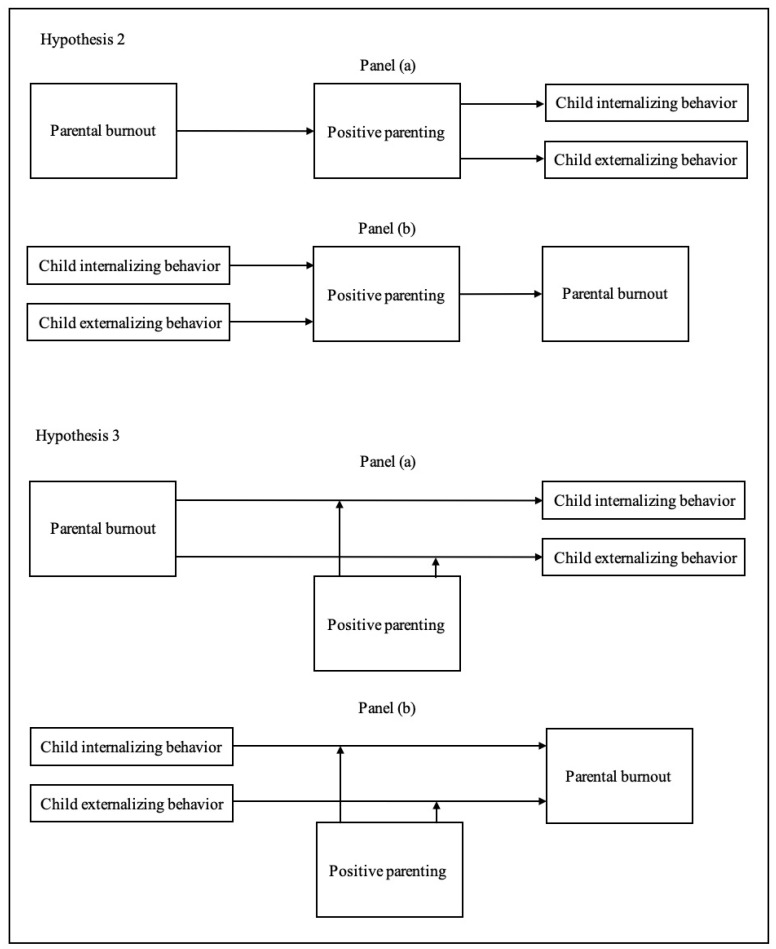
Conceptual models (simplified versions) of the posited mediating (Hypothesis 2) and moderating (Hypothesis 3) effects of positive parenting in the associations between parental burnout and child internalizing/externalizing behaviors. Panels (a) designate the mediating and moderating processes of positive parenting between parental burnout and child internalizing/externalizing behaviors, and Panels (b) designate the mediating and moderating processes of positive parenting between child internalizing and externalizing behaviors and parental burnout. Note: For the sake of parsimony, additional paths representing the direct effects in the mediation analyses were not included in the conceptual models, although they were estimated in the analyses.

Our hypotheses (conceptual models for Hypotheses 2 and 3 are graphically presented in Figure 1) unfold as follows:

**Hypothesis** **1.** *We expect bivariate correlation coefficients between parental burnout and child externalizing/internalizing behaviors to be positive and to range from weak to moderate.*

**Hypothesis** **2.** *We expect that positive parenting will mediate the association between parental burnout and child externalizing/internalizing behaviors (or between child externalizing/internalizing behaviors and parental burnout since an association does not presuppose a direction).*

**Hypothesis** **3.** *We expect that positive parenting will moderate the association between parental burnout and child externalizing/internalizing behaviors (or between child externalizing/internalizing behaviors and parental burnout since an association does not presuppose a direction).*

## 2. Method

### 2.1. Participants

In total, 383 Chilean mothers enrolled in the study. Table 1 shows the sociodemographic data of the participants. The sample size was determined using the G*Power software 3.1 [42] for correlational analyses and the R package “https://sempower.shinyapps.io/sempower/” (accessed on 12 March 2023) for the structural equation modeling analyses (mediation and moderation analyses). Respondents were invited to participate in this online study through social networks and word of mouth so as to yield a snowball sample. The survey was hosted on the PsyToolkit platform [43]. Data collection occurred between April 2021 and April 2023 in Chile. The study was presented to participants as designed to understand how exhaustion in the parenting role might be associated with child behavior. Before enrolling in the study, respondents were required to sign an online informed consent form, which covered important ethical aspects of the study. The inclusion criteria for participation in this study were as follows: both parents had to live in the same household, have at least one child between the ages of 3 and 5 (considered to be preschool age) and reside in Chile. Exclusion criteria included having adopted children with a history of early institutionalization, having children with chronic illnesses and being a parent previously diagnosed with depression.

### 2.2. Procedure

Participants first provided information about their sociodemographic situation. Second, they completed the validated Spanish version of the Parental Burnout Assessment scale (PBA, [29], Spanish version [44]). Thirdly, respondents provided an assessment of their child’s externalizing and internalizing behaviors using the Spanish version of the Strengths and Difficulties Questionnaire© (SDQ©, [45]). In a fourth case, the validated Spanish version of the Positive Parenting Scale v2.0. (E2P, [46]) was proposed to the participants. Several other instruments were used in this study, but they are not relevant to the focus of this research. These instruments included the validated Spanish version of the Interpersonal Reactivity Index (IRI, [47]; Spanish version [48]) and the validated Spanish version of the Perceived Stress Scale (PSS, [49]; Spanish version [50]), and the following instruments were administered in a synchronous online session: the Mini-Sea [51] and the validated Spanish version of the BRIEF-P ([52]; Spanish version [53]). Additionally, since data collection began one year after the outbreak of the COVID-19 pandemic, we investigated emotion regulation strategies in the presence of COVID-19, which has become an integral part of everyone’s reality. Note that the IRI, PSS, Mini-Sea and BRIEF-P instruments are not relevant to the objectives of the present study and are therefore not described in detail in the Measures section below.

The study was approved by the Institutional Review Board and was conducted in accordance with the 1964 Helsinki Declaration and its subsequent amendments.

### 2.3. Design

This exploratory correlational study, which we preregistered before accessing the data, attempts to shed light on the existence of an association between parental burnout and child behavior, which was assessed using Pearson correlation analyses (see Section 2.5.1 below). Structural equation modeling analyses were also used to test for the existence of mediating and/or moderating effects (i.e., positive parenting) in the hypothesized association between parental burnout and child behavior.

### 2.4. Measures

#### 2.4.1. Sociodemographic Questions

The survey collected data on the following sociodemographic characteristics of the female participants: age, highest educational attainment, employment status, number of working hours per week, family income level, number of hours spent with children daily, number of children living in the household, age and sex of the children.

#### 2.4.2. Parental Burnout

Parental burnout was measured using of the validated Spanish version of the Parental Burnout Assessment (PBA, [29]; Spanish version [44]), a 23-item self-report questionnaire to assess the four core symptoms of parental burnout: emotional exhaustion (9 items) (e.g., *I’m so tired out by my role as a parent that sleeping doesn’t seem like enough*), emotional distancing from one’s children (3 items) (e.g., *I do what I’m supposed to do for my child(ren) but nothing more*), loss of pleasure in one’s parental role (5 items) (e.g., *I don’t enjoy being with my child(ren)*), and contrast with previous parental self (6 items) (e.g., *I am ashamed of the parent I have become*). The items of the *PBA* use a Likert scale that ranges from 0 to 6 (viz. “never”, “a few times a year”, “once a month or less”, “a few times a month”, “once a week”, “a few times a week”, “every day”). By summing the scores of each item of the *Parental Burnout Assessment*, it is possible to calculate an individual’s parental burnout global score, which theoretically ranges from 0 to 138. A higher score indicates a higher level of parental burnout. Reliability in the present sample was assessed using Cronbach’s alpha (α = 0.96).

#### 2.4.3. Child Behavior

The respondent-rated Spanish version of the behavioral screening questionnaire, the Strengths and Difficulties Questionnaire© (SDQ©, [45]), asks about 25 positive and negative attributes of the child, thus taking into account both the child’s strengths and difficulties. It consists of 5 subscales (viz. emotional symptoms, conduct problems, hyperactivity/inattention, peer relationship problems, prosocial behavior), each with 5 items that can be marked “not true”, “somewhat true” or “certainly true”. The sum of the scores for the 5 items included in each of the 5 subscales gives a score ranging from 0 to 10. Although not relevant to this study, it is possible to obtain an overall difficulty score (ranging from 0 to 40) by summing the scores of four of the five subscales (i.e., emotional symptoms, conduct problems, hyperactivity/inattention, peer relationship problems). Note that the prosocial behavior subscale is not included in this overall difficulty score, as the absence of prosocial behavior does not necessarily imply the presence of psychological difficulties. However, what is relevant to our study is to compute a score of the child’s externalizing behavior and a second score of the child’s internalizing behavior. Summing the scores obtained in the two “conduct problems” and “hyperactivity/inattention problems” subscales allows us to generate the child’s externalizing behavior score, and similarly, summing the scores obtained in the two “emotional symptoms” and “peer relationships problems” subscales allows us to generate the child’s internalizing behavior score. Some examples of items used to rate the child’s externalizing behavior are as follows: *Generally obedient, usually does what adults request* (conduct problems subscale), *Restless*, *overactive, cannot stay still for long* (hyperactivity/inattention subscale). Some examples of items used to rate the child’s internalizing behavior are as follows: *Many worries, often seems worried* (emotional symptoms subscale)*, Picked on or bullied by other children* (peer relationship problems subscale). In this study, the version of the *SDQ©* used was that for parents whose child was between 3 and 5 years old. Reliabilities in the present sample were examined in terms of Cronbach’s alpha: α _SDQ©_externalizing behavior_ = 0.74; α _SDQ©_internalizing behavior_ = 0.56 (cf. Section 4.1).

#### 2.4.4. Positive Parenting

The validated Spanish version of the Positive Parenting Scale v2.0. (E2P) [46] is a self-report questionnaire consisting of 60 statements about daily parenting practices. Each statement is rated on a 5-point Likert scale (“never”, “almost never”, “sometimes”, “almost always” and “always”) and should be rated in relation to the last three months. The assessment tool comprises four subscales, namely parents’ relational, formative, reflective and protective competencies. Some examples illustrating the items used to assess the four types of parental competencies are as follows: Relational competencies: *I help my child calm down when he/she is stressed (crying because he/she is sick, frustrated because he/she had a problem at school, losing a game, etc.).* Formative competencies: *I invite my child to collaborate in carrying out daily activities at home (tidying up toys, setting the table, watering plants, etc.)*. Protective competencies: *When I am not with my child, I am sure that the adult or adults who care for him/her treat him/her well*. Reflective competencies: *I reflect on whether the parenting practices I use with my child are appropriate for his/her age.* The overall positive parenting score increases with the reported frequency of the parenting practice. In this sample, reliability was assessed using Cronbach’s alpha (α = 0.93).

### 2.5. Statistical Analyses

All statistical analyses were conducted using Stata version 17 [54].

#### 2.5.1. Preliminary Analyses

The Skewness and Kurtosis values were as follows: parental burnout (Skewness = 0.95; Kurtosis = 3.27), positive parenting (Skewness = −0.62; Kurtosis = 2.89), child’s internalizing behavior (Skewness = 0.63; Kurtosis = 2.85), child’s externalizing behavior (Skewness = 0.19; Kurtosis = 2.90). As none of the variables examined in this study exceeded the established threshold of |2| for Skewness and |7| for Kurtosis [55], we performed our analyses on the original (non-normalized) data. The means, standard deviations and minimum and maximum scores related to parental burnout, positive parenting and the child’s internalizing and externalizing behaviors are presented in Table 1.

In total, six participants displayed missing values on the whole child behavior measure and were therefore removed from our dataset. We subsequently observed 4 univariate outliers, which we discarded from our dataset due to implausible values. As expected, it appeared that the child’s internalizing behavior was not strongly correlated (viz. *r* = 0.27) with the child’s externalizing behavior. As the two types of behavior are obviously quite different, we decided to treat them separately in all our analyses.

### 2.6. Main Analyses

To test Hypothesis 1, we observed the bivariate correlation coefficients between parental burnout and child internalizing/externalizing behaviors in our Chilean sample. We compared the strength of these coefficients with those obtained by [12,27,28] to assess whether their findings could be replicated in our study.

To the extent that associations (of at least 0.10 [41]) between parental burnout and child internalizing/externalizing behaviors were observed in our Chilean sample, we proceeded to test Hypothesis 2. Therefore, we ran two mediation models with positive parenting as the mediator. The first model tested the mediating effect of positive parenting in the association between parental burnout and child internalizing/externalizing behaviors. Concretely, we sought to test whether parental burnout would account for less positive parenting, which in turn would lead to the child’s internalizing/externalizing behaviors. The second model reversed the direction, thus testing the effect of positive parenting in the relationship between child internalizing/externalizing behaviors and parental burnout. To put it differently, we sought to test whether the child’s internalizing/externalizing behaviors would account for less positive parenting, which in turn would lead to more parental burnout.

Finally, we tested Hypothesis 3 in which positive parenting was tested as a moderator of the associations considered in Hypothesis 2. Specifically, we were interested in finding out whether a burnt-out parent would (would not) expose their child to internalizing/externalizing behaviors if they scored low (high) on positive parenting. As for the reverse direction, we were interested in investigating whether a burnt-out parent’s engagement in much (or little) positive parenting would represent a protective (or aggravating) factor when their child displayed internalizing/externalizing behaviors. Before investigating these two moderation models, we mean-centered our variables of interest to avoid problems of multicollinearity between the main effects and the interaction term [56].

## 3. Results

**Hypothesis** **1.** *We expect bivariate correlation coefficients between parental burnout and child externalizing/internalizing behaviors to be positive and to range from weak to moderate.*

The results related to this first hypothesis are presented in Table 2. As opposed to the child’s internalizing behavior, which was weakly correlated with parental burnout (*r* = 0.12), the size of the bivariate correlation coefficient between the child’s externalizing behavior and parental burnout was moderate (*r* = 0.32). Our results thus replicate those of the authors of [12,27,28], who observed correlation coefficients between parental burnout and child internalizing/externalizing problems ranging from 0.12 to 0.24 in their respective samples. Additionally, as in Chen and his colleagues’ study [12], we observed that the association between parental burnout and child externalizing behavior was statistically stronger than the association between parental burnout and child internalizing behavior, *z* = −2.91, *p* < 0.01.

With our first hypothesis thus validated, we proceeded to test our mediation and moderation hypotheses.

**Hypothesis** **2.** *We expect that positive parenting will mediate the association between parental burnout and child externalizing/internalizing behaviors (or between child externalizing/internalizing behaviors and parental burnout since an association does not presuppose a direction).*

Table 3 provides full information on the direct, indirect and total effects of the two mediation models tested. With regard to the relationship between parental burnout and the child’s internalizing/externalizing behaviors via positive parenting (Panel (a)), we observed that positive parenting (i) fully mediated the relationship between parental burnout and the child’s internalizing behavior (direct effect: β = 0.01, *p >* 0.05; indirect effect: β = 0.04, *p <* 0.01) and (ii) partially mediated the relationship between parental burnout and the child’s externalizing behavior (direct effect: β = 0.03, *p <* 0.001; indirect effect: β = 0.01, *p <* 0.01) with a direct negative effect of parental burnout on positive parenting (β = −0.21, *p <* 0.001) suggesting that a parent dealing with parental burnout would be less likely to engage in positive parenting. In turn, positive parenting appeared to be related to the child’s internalizing and externalizing behaviors. While the child’s internalizing behavior accounted for 3% of the variance explained in the model, the child’s externalizing behavior accounted for 13%.

Now considering the reverse direction, that is, the association between the child’s internalizing/externalizing behavior and parental burnout via positive parenting (Panel (b)), we observed that positive parenting only partially mediated the relationship between the child’s externalizing behavior and parental burnout (direct effect: β = 1.95, *p* < 0.001; indirect effect: β = 0.36, *p* < 0.01) with a direct negative effect of the child’s externalizing behavior on positive parenting (β = −0.86, *p* < 0.05), suggesting that a child, by being unruly, aggressive or defiant, might reduce the occurrence of positive parenting in their parents. In turn, positive parenting appeared to be related to parental burnout. Parental burnout accounted for 13% of the variance explained in the model.

If the results regarding the relationship between parental burnout and the child’s internalizing/externalizing behaviors fully support our Hypothesis 2, those pertaining to the link between the child’s internalizing/externalizing behaviors and parental burnout only partially support it, as the relationship between the child’s internalizing behavior and parental burnout was not mediated by positive parenting.

**Hypothesis** **3.** *We expect that positive parenting will moderate the association between parental burnout and child externalizing/internalizing behaviors (or between child externalizing/internalizing behaviors and parental burnout since an association does not presuppose a direction).*

All statistically (non)significant main effects and interaction terms are listed in Table 4. Regarding the hypothesized moderating effect of positive parenting in the association between parental burnout and the child’s internalizing/externalizing behaviors (Panel (a)), we observed three main effects as follows: (i) one main effect of positive parenting (β = −0.21, *p* < 0.05) on the child’s internalizing behavior; (ii) two main effects of both parental burnout (β = 0.26, *p* < 0.001) and positive parenting (β = −0.24, *p* < 0.01) on the child’s externalizing behavior. No interaction term was observed. While the child’s internalizing behavior accounted for 4% of the variance explained in the model, the child’s externalizing behavior accounted for 13%.

As for the hypothesized moderating effect of positive parenting in the relationship between the child’s internalizing/externalizing behaviors and parental burnout (Panel (b)), we observed two main effects as follows: (i) a main effect of the child’s externalizing behavior (β = 0.25, *p* < 0.001) and a second main effect of positive parenting (β = −0.25, *p* < 0.05) on parental burnout. No interaction was observed. Parental burnout accounted for 14% of the variance explained in the model.

These results unequivocally invalidate our Hypothesis 3 since no interaction effect could be observed.

## 4. Discussion

The present study showed that the associations between parental burnout and child externalizing/internalizing behaviors, previously observed in Chinese settings with adolescents ([12,27,28]), could be replicated with measures taken in South America (Chile) among preschool children. In addition to replicating the existence of these associations, our study also confirmed a stronger association between parental burnout and externalizing (as opposed to internalizing) child behavior.

The cross-sectional study further explored mediating/moderating effects of positive parenting in the bivariate association (precluding any causal direction) between parental burnout and child behavior, or between child behavior and parental burnout. It highlighted the role of positive parenting as a mediator in the relationship linking parental burnout and the child’s internalizing (full mediation) and externalizing (partial mediation) behaviors. The full mediating effect of positive parenting in this relationship suggested that parental burnout alone would not put the child at risk of developing internalizing behavioral problems. Instead, parental burnout would reduce the occurrence/quality of positive parenting, which in turn might have a detrimental effect on the child’s internalizing behavior. In a nutshell, parental burnout would not directly relate to the likelihood of a child exhibiting internalizing behavior. However, if parental burnout were to lead to a deterioration in positive parenting, then, it might impact the child’s internalizing behavior. This finding dovetails with Rose et al.’s [25] systematic review which highlights that poor positive parenting relates to the child’s internalizing behavior. As for the partial mediating effect of positive parenting in the association between parental burnout and the child’s externalizing behavior, it appeared that parental burnout would not only have a detrimental effect on the child’s externalizing behavior, but also reduce the occurrence/quality of positive parenting, which in turn may have a detrimental effect on the child’s externalizing behavior. The observed deleterious effect of parental burnout on positive parenting, possibly leading in turn to child behavior issues, is echoed by Chen et al. [12], who show that maternal hostility mediates the relationship between parental burnout and internalizing/externalizing behaviors in the offspring.

Still regarding the mediating effects of positive parenting, but this time looking at the reverse direction—that is, from the child’s internalizing/externalizing behaviors to parental burnout—we observed that positive parenting only partially mediated the relationship between the child’s externalizing behavior and parental burnout. In other words, the child’s externalizing behavior would not only contribute to parental burnout, but it would also reduce the occurrence/quality of positive parenting, which in turn might predispose the parent to more parental burnout. Aligning with the idea of the impact that the child’s externalizing behavior may have on their parent, Larsson et al. [39] showed that the child’s antisocial behavior deleteriously affected the parent. Serbin et al. [30] further longitudinally showed that the child’s externalizing behavior predicted reduced levels of positive parenting. By contrast, we did not observe a mediating effect of positive parenting between the child’s internalizing behavior and parental burnout. An attempt to explain this observation is given below when we discuss the discrepancies observed in the explained variances between the child’s internalizing and externalizing behaviors.

Surprisingly, regardless of the direction (i.e., from parental burnout to child internalizing/externalizing behaviors, or from the child’s internalizing/externalizing behaviors to parental burnout), no moderating effect of positive parenting could be observed in the associations. If we translate this finding in terms of risk perception, this would mean that the likelihood of parental burnout potentially leading to child internalizing/externalizing behaviors would not be related to the average level of positive parenting exhibited by the parent. The absence of a moderating effect of positive parenting in our study does not necessarily imply that moderating effects are less likely than mediating effects in the observed associations, but rather that we are possibly faced with an artifact since a majority of participants scored fairly high on positive parenting (idea of ceiling/floor effects; see Table 1 for descriptive statistics of the positive parenting variable). This artifact appears even more plausible since positive parenting is a socially desirable trait particularly valued by mothers from educated backgrounds who were over-represented in our sample. Another explanation for this may lie in the fact that it would be fanciful to imagine that a parent who is burnt out can find the energy to engage in positive parenting to buffer the deleterious consequences of their syndrome.

Noteworthily, the observed discrepancy between the proportion of explained variance attributed to the child’s internalizing behaviors (i.e., 3% in the mediation model and 4% in the moderation model) and that ascribed to the child’s externalizing behavior (i.e., 13% in both the mediation and the moderation models) might indicate that the child’s externalizing behavior may be a more substantial contributing factor to parental burnout than the child’s internalizing behavior might be. This makes perfect sense if we take the perspective of the burnt-out parent. Indeed, one possible explanation for this discrepancy could be that a defiant, aggressive or oppositional child (externalizing behavior) probably causes more worry/burden to their exhausted parent already considerably weakened by their parental burnout symptoms, whereas a more discreet child—withdrawn into their inner world (internalizing behavior)—probably causes less (if any) worry/burden to their exhausted and therefore detached parent. However, it is possible that the low reliability indices of the scale used to measure the child’s internalizing behavior may be the cause of this discrepancy. Please refer to the ‘Measures’ and ‘Limitations and Future Directions’ sections for more information.

### 4.1. Limitations and Future Directions

Although promising in the field of parental burnout, the present study is not without its shortcomings. The psychometric properties of the “internalizing behavior” subscale of the Strengths and Difficulties Questionnaire© [45] were poor (α = 0.56). The possibility of removing one (or several) of the items to improve the alpha was considered but ultimately rejected as this would have worsened the psychometric properties.

Most importantly, the current results based on a correlational method are not sufficient to allow the conclusion of a bidirectional relationship between parental burnout and child internalizing and externalizing behaviors or the conclusion of the existence of mediating/moderating effects such as positive parenting in the association. Thus, the employed design prevents both the generalizability of the results and the identification of causal pathways. In this respect, the present endeavor calls both for caution in the interpretation of the results and for further studies that would test similar hypotheses using a cross-lagged panel model method.

The advantages of testing the association between parental burnout and child behavior in a longitudinal model are manifold. First, it would make it possible to determine whether the associations observed here actually exist or whether, for example, unidirectional or transactional effects should rather be considered. Second, the use of a longitudinal model would make it possible to quantify the proportion of the effect attributable to the parent and that attributable to the child. In other words, this would enable us to figure out whether it is parental burnout that is more at the origin of behavioral issues in the child, or whether it is rather the child’s behavioral issues that are more at the origin of parental burnout—or both equally. Thirdly, and following on from the above, such a study design would also allow us to gain a more precise insight into the potential causal pathways between the child’s internalizing and externalizing behaviors specifically and parental burnout. This question seems all the more relevant as the models tested here tended to show that the child’s externalizing behavior explained more variance in comparison to its internalizing counterpart. Finally, since the use of a cross-lagged panel model would make it possible to identify both the direction and the parent–child share of the effect, once these effects are known, it would be possible to test mediating effects (which, in the present study, proved more convincing than moderating effects) of specific variables that would make sense in the (bidirectional) relationship that would have been established by the cross-lagged panel model.

## 5. Conclusions

The present research aimed at exploring the mediating and moderating effects of positive parenting in the hypothesized bivariate associations between parental burnout and child internalizing and externalizing behaviors. Contrary to moderating effects, mediating effects were observed in the associations. Thus, positive parenting mediated the association between parental burnout and the child’s internalizing (full mediation) and externalizing (partial mediation) behaviors. Positive parenting also partially mediated the association between the child’s externalizing behavior and parental burnout. We hope that this initial cross-sectional study will provide considerable impetus to scholars wishing to delve deeper into the under-researched topic of the putative bidirectional relationship between child behavior and parental burnout.

## Figures and Tables

**Table 1:** Sociodemographic characteristics of the participants and mean, standard deviation, minimum and maximum of the variables of interest.

Variables	%	M	SD	Min	Max
Age		35.78	5.61	23	57
Educational attainment					
Incomplete basic education or less	0.53				
Complete elementary school	2.11				
High school incomplete	10				
High school complete	21.84				
University incomplete	46.84				
University complete	18.68				
Post-graduate (Master’s, PhD or equivalent)					
Paid work status					
Yes (vs. No)	68.93				
Number of hours per week		24.04	18.78	6	100
Total monthly household income					
Less than CLP 500,000	11.02				
Between CLP 501,000 and CLP 1,200,000	32.26				
CLP 1,201,000 and above	56.72				
Number of hours spent with children daily		12.57	7.71	2	24
Age of children		4.05	1.57		
Number of children		1.73	0.89		
Female children	56.46				
Strengths and Difficulties Questionnaire© (internalizing behavior)		3.31	2.39	0	11
Strengths and Difficulties Questionnaire© (externalizing behavior)		7.31	3.26	0	19
Positive Parenting Scale (total score)		205.90	20.01	144	240
Parental Burnout Assessment (total score)		34.52	25.78	0	116

**Table 2:** Bivariate relations between parental burnout, child internalizing behavior and child externalizing behavior.

Variables		1	2	3	4
1	Parental burnout	−			
2	Positive parenting	−0.27 ***	−		
3	Child internalizing behavior	−0.12 *	−0.17 ***	−	
4	Child externalizing behavior	−0.32 ***	−0.26 ***	0.27 ***	−

Note: * *p* < 0.05, *** *p* < 0.001.

**Table 3:** Direct, indirect and total effects in the associations between parental burnout and child internalizing and externalizing behaviors.

Panel (a): Parental Burnout → Child Internalizing/Externalizing Behaviors									
	Direct Effect			Indirect Effect			Total Effect		
Path	β	95% CI	*SE* β	β	95% CI	*SE* β	β	95% CI	*SE* β
Parental burnout → positive parenting	−0.21 ***	[−0.28, −0.13]	0.04	−	−	−	−0.21 ***	[−0.28, −0.13]	0.04
Positive parenting → internalizing behavior	−0.02 **	[−0.03, −0.01]	0.01	−	−	−	−0.02 **	[−0.03, −0.01]	0.01
Positive parenting → externalizing behavior	−0.03 ***	[−0.05, −0.02]	0.01	−	−	−	−0.03 ***	[−0.05, −0.02]	0.01
Parental burnout → internalizing behavior	0.01	[−0.002, 0.02]	0.004	0.004 **	[0.001, 0.01]	0.001	0.01 **	[0.001, 0.02]	0.004
Parental burnout → externalizing behavior	0.03 ***	[0.02, 0.05]	0.01	0.01 **	[0.002, 0.01]	0.002	0.04 ***	[0.03, 0.05]	0.01
R^2^	0.14 ***								
**Panel (b): Child Internalizing/Externalizing Behaviors** **→ Parental Burnout**									
	**Direct Effect**			**Indirect Effect**			**Total Effect**		
**Path**	**β**	**95% CI**	***SE* β**	**β**	**95% CI**	***SE* β**	**β**	**95% CI**	***SE* β**
Internalizing behavior → positive parenting	−0.86 *	[−1.69, −0.03]	0.42	−	−	−	−0.86 *	[−1.69, −0.03]	0.42
Externalizing behavior → positive parenting	−1.40 ***	[−1.99, −0.79]	0.31	−	−	−	−1.40 ***	[−1.99, −0.79]	0.31
Positive parenting → parental burnout	−0.26 ***	[−0.38, −0.13]	0.06	−	−	−	−0.26 ***	[−0.38, −0.13]	0.06
Internalizing behavior → parental burnout	0.13	[−0.92, 1.17]	0.53	0.22	[−0.02, 0.46]	0.12	0.35	[−0.71, 1.41]	0.54
Externalizing behavior → parental burnout	1.95 ***	[1.17, 2.72]	0.40	0.36 **	[0.12, 0.59]	0.12	2.30 ***	[1.53, 3.07]	0.39
R^2^	0.14 ***								

Note: * *p* < 0.05. ** *p* < 0.01. *** *p* < 0.001. Panel (a) designates the mediational process of positive parenting between parental burnout and child internalizing/externalizing behaviors, and Panel (b) designates the mediational process of positive parenting between child internalizing and externalizing behaviors and parental burnout.

**Table 4:** Moderating effects of positive parenting in the associations between parental burnout and child internalizing/externalizing behaviors.

Panel (a)				
Parental Burnout → Child Internalizing Behavior			95% CI for β	
Main Effects	β	SE	Lower	Upper
Parental burnout	0.08	0.05	−0.02	0.18
Positive parenting	−0.21 *	0.09	−0.38	−0.04
**Interaction term**				
Parental burnout × positive parenting	0.08	0.09	−0.09	0.25
**Parental burnout** **→ Child externalizing behavior**			**95% CI for β**	
**Main effects**	**β**	**SE**	**Lower**	**Upper**
Parental burnout	0.26 ***	0.05	0.16	0.35
Positive parenting	−0.24 **	0.08	−0.40	−0.08
**Interaction term**				
Parental burnout × positive parenting	0.06	0.08	−0.10	0.22
**Panel (b)**			**95% CI for β**	
**Main Effects**	**β**	**SE**	**Lower**	**Upper**
Internalizing behavior	0.01	0.05	−0.09	0.11
Externalizing behavior	0.25 ***	0.05	0.16	0.35
Positive parenting	−0.25 *	0.12	−0.49	−0.01
**Interaction terms**				
Internalizing behavior × positive parenting	0.09	0.09	−0.09	0.26
Externalizing behavior × positive parenting	−0.02	0.13	−0.27	0.23

Note: * *p* < 0.05. ** *p* < 0.01. *** *p* < 0.001. Coefficients are standardized. Panel (a) designates the moderating process of positive parenting between parental burnout and child internalizing/externalizing behaviors, and Panel (b) designates the moderating process of positive parenting between child internalizing and externalizing behaviors and parental burnout. *R*^2^_panel (a)_ = 0.14 ***; *R*^2^_Panel (b)_ = 0.13 ***.

## Data Availability

The analysis plan, methodology and a description of the dataset were preregistered on the Open Science Framework (OSF). The data presented in this study are openly available on OSF at https://osf.io/5urt8/, accessed on 15 March 2024.
